# Planning of physiotherapeutic procedure in patients after mandible reconstruction taking into account donor site: a literature review

**DOI:** 10.1186/s40001-023-01386-y

**Published:** 2023-09-28

**Authors:** Julia Walatek, Andrzej Myśliwiec, Łukasz Krakowczyk, Wojciech Wolański, Anna Lipowicz, Krzysztof Dowgierd

**Affiliations:** 1 Department of Science, Innovation and Development, Galen-Orthopedics, 43-150 Bierun, Poland; 2 Laboratory of Physiotherapy and Physioprevention, Institute of Physiotherapy and Health Sciences, Academy of Physical Education, 40-065 Katowice, Poland; 3 Department of Oncologic and Reconstructive Surgery, Maria Sklodowska-Curie National Research Institute of Oncology, 44-102 Gliwice, Poland; 4 Department of Biomechatronics, Faculty of Biomedical Engineering, Silesian University of Technology, 41-800 Zabrze, Poland; 5 Department of Anthropology, Institute of Environmental Biology, Wroclaw University of Environmental and Life Sciences, 50-375 Wroclaw, Poland; 6 Head and Neck Surgery Clinic for Children and Young Adults, Department of Clinical Pediatrics, University of Warmia and Mazury, 10-561 Olsztyn, Poland

**Keywords:** Mandible, Tumor, Reconstruction, Physiotherapy, Donor site, Transplantology, Oncology, Rehabilitation

## Abstract

**Background:**

Mandible tumors are very rare. One of the main methods of the treatments is resection of the tumor and then reconstruction of the mandible. The donor site is often distant tissue—fibula or ilium. Following this, it is necessary to improve the patient in two ways, on one hand restoring the function of the mandible, and on the other hand, improving the donor site area. For that reason, physiotherapy after tumor resection and reconstruction of the mandible is very complicated. The aim of this bibliographic review was to find the methods of the reconstruction of the mandible in the context of patients’ functional assessment after surgeries to create effective physiotherapeutic procedures in the feature.

**Methods:**

PEDro, Medline (PubMed), Cochrane Clinical Trials were searched.

**Results:**

767 articles were found. 40 articles were included to this literature review.

**Conclusions:**

Authors showed different kinds of surgeries strategy for patients with tumors of the mandible. They also showed manners of patients’ functional assessment in the localization of transplantation and donor site. It could be useful for physiotherapists during planning of comprehensive physiotherapy.

## Introduction

Primary tumors are one of the rarest and enhance 3% of all bone tumors [1]. They are also a small percentage of all cancers in the pediatric population. One should pay attention that their development is connected with bone growth retardation, appearing big deformation of the face, and also the necessity of conducting of complicated surgeries with the transplantation of tissues simultaneously [2]. The location of odontogenic tumors such as odontoma or ameloblastoma is most often the interior of the jaw bone or the surrounding soft tissues [1, 3, 4]. These tumors are formed from inactive cells forming the teeth (enamel, dentine, pulp) and jaw bones and the moment of their formation may coincide with the development of the oral cavity [4]. On the other hand, non-odontogenic tumors, such as central giant cell granulomas or fibro-osseous lesions, are formed from bone or mesenchymal tissue and are often malignant [3–5]. The etiology of mandibular tumors remains unknown, which often makes accurate diagnosis difficult [1, 5]. Currently, the most commonly used and most effective method of treating pathological changes in the mandible is its partial resection with simultaneous reconstruction in the form of free flaps most often fibula or the wing of ilium [6–8]. In the case of malignant tumors, adjuvant radiotherapy or chemotherapy is also used [7]. Reconstructive surgery brings not only benefits such as the reconstruction of the removed part of the mandible but is also burdened with some risks and complications resulting from the collection of tissues from other areas of the body. In the case of taking a graft from the fibula, patients often report pain and sensory disturbances at the donor site, as well as a feeling of ankle instability [9, 10]. In some patients, a clear decrease in gait speed, contracture of the flexor hallucis longus, limitation of the range of motion of the dorsal and plantar flexion in the ankle joint, as well as muscle weakness or contracture of the toes can be seen [9, 11, 12]. On the other hand, people undergoing reconstruction with iliac crest free flap collection struggle with limitation of the range of motion in the hip joint or stiffness of the Achilles tendon [13, 14]. The aim of the study was to review the methods of mandibular reconstruction with particular emphasis on the functional assessment after transplantation, both in the context of the mandible function and the function of the donor site, which may be the basis for future programming of physiotherapeutic procedures. Attention was paid to the possible effects of transplants and their impact on the possibilities of rehabilitation procedures.

## Materials and methods

**Fig. 1:**
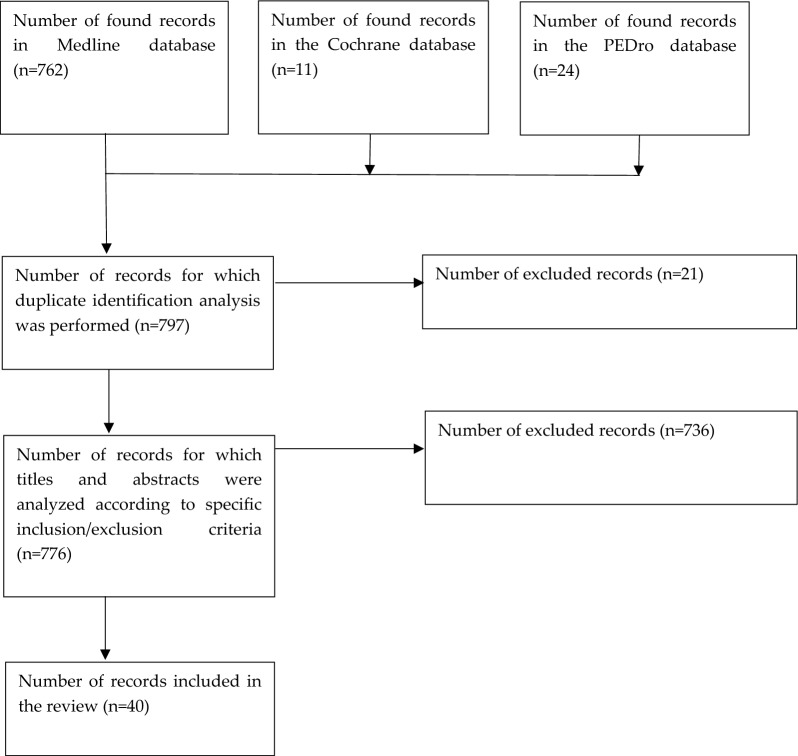
Summary of the search results

PEDro, Medline (PubMed), Cochrane Clinical Trials database were searched. Different combinations of the keywords were used: mandibular cancer, mandibular tumor, mandibular tumour, jaw cancer, jaw tumor, jaw tumour, treatment, therapy, reconstruction, physiotherapy, physical therapy, scar, fibula free flap, costal graft, rib graft, scapula graft, occlusion, complication, gait, assessment, evaluation, function. The research started with the main phrase: ((treatment OR therapy) AND (child* OR pediatric*) AND ((mandibular cancer*) OR (mandibular tumor*) OR (mandible cancer*) OR (mandible tumor*) OR (jaw* cancer*) OR (jaw* tumor*))). Then, due to the small number of publications found, additional phrases were used: ((mandible) AND (reconstruction) AND (physiotherapy)), (jaw) AND (reconstruction) AND (physiotherapy)), ((physical therapy) AND (mandible) AND (reconstruction)), ((mandible reconstruction) AND (scar), ((free flap iliac) AND (mandibula*)), ((rib graft) AND (mandibula*)), ((costal graft) AND (mandibula*)), ((scapula graft) AND (mandible*)), ((costal graft) AND (physiotherapy)), ((rib graft) AND (physiotherapy)), ((scapula graft) AND (physiotherapy)), ((free flap) AND (physiotherapy)), ((occlusion) AND (mandible reconstruction)), ((complication) AND (mandible reconstruction)), ((gait) AND (mandible reconstruction)), ((gait) AND (free flap)), ((mandible reconstruction) AND ((assessment) OR (evaluation))), ((mandible reconstruction) AND (function)). Due to the significantly limited number of publications related to the topic above and the high heterogeneity of research, the qualitative assessment of the found publications was omitted. Inclusion criteria were clinical trials and case studies concerning mandibular reconstruction methods in populations where the majority of the patients had oncological diagnosis within the mandible. Selected studies could use any surgical protocols during reconstruction procedures, as well as any method of assessing the patient's postoperative condition, including short-term and/or long-term effects. Articles in Polish and English, which were published in the years 2015–2022, were analyzed. All the articles which were meta-analyses, systematic reviews, included non-autologous transplants, did not contain postoperative and/or short-term and/or long-term assessment of the patient's condition were excluded. Figure 1 summarizes the search results.

## Results

40 articles were qualified for the bibliographic review. They described the surgical procedure, the function of the mandible, mouth, speech apparatus, esophagus assessed by the researchers, as well as the function associated with the donor site and in some cases other ailments accompanying patients after mandibular reconstruction. The most frequently mentioned methods of mandibular reconstruction in the literature were those using fibula free flap and iliac crest free flap. A detailed summary of the included articles is in Tables 1, 2, 3.

**Table 1:** Articles in which only jaw and/or mouth and/or the apparatus of speech and/or esophagus function was evaluated

No.	Author and year	Disease	P	Donor site	Evaluation of the jaw and/or mouth and/or the speech apparatus and/or esophagus function
1.	Abramowicz et al. [30]	AB, DF, OF, KCOT, fibromyxoid sarcoma, odontogenic cyst	*n* = 15 age 9–17	FFF	Speech assessment and the need for eating soft food
2.	Askin et al. [31]	AB	*n* = 1 age 29	ICFF	Full satisfaction with the function of the mandible
3.	Bouchet et al. [15]	SCC, sarcoma	*n* = 25 age 29–77	FFF	Bite strength 50–462 N, possibility of chewing 1–6 on a scale, laterotrusion 2.3–3 mm, protrusion 1.8–2.1 mm, mouth opening 3.1–3.5 cm, satisfaction with the function of chewing and eating food 1–10 (VAS), quality of life 1–5 on a scale in different spheres of life
4.	Comini et al. [32]	OS, ES	*n *= 4 age 7–14	CG, FFF, fibular epiphyseal flap	Lack of major impairments of speech, phonation, chewing, breathing
5.	Dowgierd et al. [27]	Fibroma ossificans, sarcoma, AB, CGCG	*n* = 9 age 8–17	ICFF, FFF	Increased mouth opening (3–4.5 cm), satisfaction or worsening of TMJ function
6.	Gravvanis et al. [16]	AB, OM	*n* = 4 age 27–47	FFF	Understandable or dysfunctional speech, normal or difficult chewing and swallowing of hard food, normal TMJ mobility
7.	Guo et al. [33]	Lower gingival cancer, ES, AB, floor of mouth cancer, OS, OM, tongue cancer, giant cell tumor	*n *= 17 age 20–64	CG, nonvascularized fibula bone graft, pectoralis major flap, FFF, nonvascularized iliac bone graft, local flap	Excellent or good chewing function with the ability to eat all or soft food, excellent or good speech function, understanding of the patient during a conversation (2.33–5 on the scale)
8.	Hu et al. [34]	AB	*n* = 1 age 31	Nonvascularized free iliac bone graft	Good chewing function, good jaw function
9.	Jarefors et al. [17]	SCC, clear cell tumor, salivary gland cancer, osteoradionecrosis, osteomyelitis	*n *= 17 age 43–79	FFF	Good or reduced range of mouth opening (2–9.5 cm), problems with the function of chewing, swallowing, speaking
10.	Johnson et al. [28]	AB, cystic lesion	*n* = 25 age 18–55	CG	Normal, understandable speech or speech with minor problems, chewing ability, no problems with eating soft foods, minor problems with salivation control, symmetrical or asymmetrical opening of the mouth for more than 3 cm
11.	Kalwagadda et al. [43]	AB	*n* = 46 age 11–85	CG, FFF	Easy understandable speech, adequate opening of the oral cavity
12.	Khachatryan et al. [35]	AB, eosinophilic granuloma, KCOT, OS	*n* = 21 age 28–63	FFF	Good chewing function, acceptable function in patients’ evaluation
13.	Khatib et al. [53]	DF, periosteal fibromatosis,	*n* = 3 age 2–9	ICFF, CG	Normal mandibular mobility
14.	Lin et al. [36]	AB, SCC, OF, OM, Langerhans cell histiocytosis, mucoepidermoid carcinoma	*n* = 10 age 32–40	nonvascularized fibula flap	No reported difficulties in speaking and swallowing
15.	Lv et al. [21]	AB, OM, OF, osteoblastoma	*n* = 51 age 21–55	FFF, ICFF	Oral opening range 3.5–4.5 cm, no difficulties in speaking and swallowing
16.	Olvera-Caballero et al. [19]	AB, osteomyelitis, DF, CGCG	*n* = 6 age 8–62	FFF, scapula osseous free flap	Mostly an excellent function, but not in all, normal chewing function preserved, absence of speaking and swallowing disorders
17.	Sakata et al. [56]	SCC	*n* = 2 age 59–70	ICFF	No complications, the need for a soft diet for a year
18.	Sakuraba et al. [22]	SCC	*n* = 101 age 24–77	FFF	Normal or soft diet, normal or impaired speech, decrease in chewing strength on the transplanted side
19.	Tarsitano et al. [37]	KCOT, SCC, osteogenic sarcoma, sarcoma, odontogenic fibromyxoma	*n* = 9 age 17–75	FFF	Possibility of eating normal, soft or liquid food, normal or reduced range of mouth opening, jaw deviation during maximum opening
20.	Tian et al. [38]	AB	*n* = 1 age 44	ICFF	Patient satisfaction with functional restoration
21.	Valentini et al. (2018) [55]	Desmoid fibromatosis, myxoid fibroma, chondroblastic OS, FD, fibromatosis, rhabdomyosarcoma, OF, CGCG, AB, KCOT, OS, plexiform sarcoma, gunshot, melanocytic neuroectodermal tumor, ES, mucoepidermoid carcinoma	*n* = 25 age 0–18	FFF, ICFF, latissimus dorsi free flap, rib graft, local flap, scapula free flap, rectus abdominis free flap, temporalis flap	Good phonation and swallowing, normal or good oral function
22.	Zhang J et al. [25]	SCC, AB	*n *= 56 age 31–65	FFF	Temporary deterioration of chewing performance in the examination and in the patients’ evaluation
23.	Zhu et al. [57]	FD	*n* = 1 age 30	FFF	No damage to facial function

**Table 2:** Articles that only evaluate the global function related to the donor site

No.	Author and year	Disease	*P*	Donor site	Evaluation of the global function related to the donor site
24.	Bachelet et al. [47]	AB, aggressive fibromatosis, giant mandibular cyst, trauma, mucoepidermoid carcinoma, osteochemonecrosis, first arch syndrome	*n* = 54 median age 31	CG	No physical and functional complications
25.	Li et al. [13]	Adenocarcinoma, melanoma, mucoepidermoid cyst, sarcoma, adenoid cystic carcinoma, carcinoma not otherwise specified, esthesioneuroblastoma, no residual tumor	n = 154 median age 57–63	FFF, ICFF	Clawing of the great toe, ankle stiffness
26.	Maben et al. [41]	SCC, SpCC, AB, OF, KCOT	*n* = 20 age 17–60	FFF	No problems with walking or problems of different degrees, difficulties in activities of daily living to different degree, change in gait pattern
27.	Rendenbach et al. [39]	Malignomas of the head and neck region	*n* = 19 age 31–74	FFF	Significant reduction of jump height and decrease in AOFAS-Score, no significant differences between limbs in maximum strength and peak power, One-Leg Balance Testing and motions in the mediolateral direction, increase in motions in the anteroposterior direction in both limbs, a significant deficit in sagittal motion in the ankle joint, reported big toe contracture, persistent numbness at the donor site, limitations of everyday activities and subjective gait insecurity
28.	Rendenbach et al. [14]	AB, SCC, osteonecrosis, myxofibroma, mucoepidermoid carcinoma	*n* = 14 age 22–87	ICFF	No significant differences before and after surgery on the Esslinger Fitness Index, chair raising test, jumping tests, balance testing, a significant reduction all ranges of motion in the lumbar spine and hip joint except for dorsal extension, occasional limitations of everyday activities (getting up from sitting or lying down, doing sports)
29.	Xu et al. [40]	SCC, AB, OM, KCOT	*n* = 30 age 20–66	FFF	Decrease in ankle isokinetic test, results of the EMG test categorized as 3 types, changes in plantar pressure distribution, reported weakness of the limb
30.	Zheng et al. [29]	Benign tumor, OS	*n* = 4 age 12–32	ICFF	No gait disturbances associated with the donor site

**Table 3:** Articles in which both the jaw and/or mouth and/or the apparatus of speech and/or esophagus function, as well as the global function associated with the donor site, were evaluated

No.	Author and year	Disease	P	Donor site	Evaluation of the function of the jaw and/or mouth and/or the speech apparatus and/or esophagus	Evaluation of the global function related to the donor site
31.	Abdelrehem et al. [42]	AB, OM, malignant tumor of jaw, KCOT	*n* = 12 age 18–57	Innervated vascularizediliac bone	No speech disorders in some patients, accidental bites on the lip, salivation 0–3.17 on a scale	No restrictions in everyday activities
32.	Devireddy et al. [26]	OF, AB, KCOT	*n* = 7 age 13–63	Nonvascular fibula graft	No restrictions, deviation in one of the directions or no large changes in the jaw movement, no restrictions on tongue mobility, limited opening or improvement of the mouth opening 3.5–4.2 cm	No foot drooping
33.	Okoturo [18]	AB, OF, fibromyxoma, recurrent odontogenic cyst, CGCG	*n* = 18 age 19–42	Nonvascularized iliac crest bone graft	Good or acceptable mandibular function	No reported complications
34.	Puricelli et al. [54]	OS	*n* = 1 age 27	FFF, ICFF, CG	Maintaining a smile, mouth opening without lateral deviation, control of salivation and chewing, small problems with speech function, no difficulties in head moving	No complications at the donor site
35.	Shahzad et al. [44]	ES, cement OF, FD, metastatic neuroblastoma, chondrosarcoma, osteogenic sarcoma, DF, osteogenic sarcoma, ameloblastic fibrosarcoma	*n* = 10 age 3–17	FFF	No changes in the quality of speaking, normal diet possible, clicking or jaw deviation during opening	Normal lower limb function, ankle pain while running, Achilles tendon contracture
36.	Yamamoto et al. [24]	Mandibular gingival carcinoma, AB, mandibular, FD, aneurysmal bone cyst, osteomyelitis	*n* = 8 age 38–74	FFF	Increase in bite strength compared to strength before surgery	No difficulties during walk
37.	Zavala et al. [20]	OF, AB, arteriovenous malformation, FD, GS, dentigerous cyst, surgical sequel of mandibular lymphoma, COF, adenomatoid odontogenic tumor, aneurysmal bone cyst, NS, CGCG	*n* = 34 age 2–15	FFF	Limitation of the mouth opening, ankylosis, the possibility of eating rated at 4–6 on a scale	Lack of big toe flexion and contracture, instability or deformity of the ankle joint, difficulties during walk
38.	Zhang C et al. [23]	SCC	*n* = 1 age 56	ICFF	Mouth opening range 2.5 cm, no tongue mobility restrictions	No hip mobility restrictions
39.	Zhang M et al. [46]	AB, OF, OM, keratosis odontogenic cyst	*n* = 20 age 19–51	ICFF	Satisfaction with chewing, TMJ function and occlusion assessed by patients	No mobility impairments
40.	Zou et al. [45]	AB, OM, SCC, KCOT	*n* = 32 age 24–61	ICFF	Fll or partial patients’ satisfaction with oral function, pronunciation, range of mouth opening, tongue mobility limitations	Slight gait disturbances

*P* population (age in years), *FFF* fibula free flap, *TM*J temporo-mandibular joint, *SCC* squamous cell carcinoma, *OS* osteosarcoma, *CGCG* central giant-cell granuloma, *KCOT* keratocystic odontogenic tumor; *OF* ossifying fibroma, *GS* Goldenhar syndrome, *COF* central odontogenic fibroma, *OM* odontogenic myxoma, *NS* neurogenic sarcoma, *FD* fibrous dysplasia, *ES* Ewing sarcoma, *SpCC* spindle cell carcinoma, *AB* ameloblastoma, *DF* desmoplastic fibroma, *ICFF* iliac crest free flap, *CG* costal graft

### Evaluation of the jaw, mouth, speech apparatus, esophagus function

In the articles selected for review, the authors most often made a functional assessment directly related to the place after mandibular reconstruction. The evaluation referred among others to the range of mouth opening, chewing, swallowing, speech quality, speech understanding and tongue mobility. In most instances, function control is based on subjective patients’ assessment using standardized scales and questionnaires such as the Functional Intraoral Glasgow Scale, Head and Neck Performance Status Scale, University of Washington Quality-of-Life, Oral Health Impact Profile, Eating Ability Evaluation Questionnaire, or 14-item Oral Health Impact Profile Questionnaire [15–20]. Methods of objective function assessment, such as measurements of the range of mouth opening or measurements of bite strength, appeared much less frequently in research [21–23]. Sakuraba et al. [22] assessed the mandible function taking into account not only the subjective feelings of the patient but also the objectified measurements using the GM10 bite force meter. This gauge was placed between the first premolar and the second molar. However, bilateral measurement comparing the healthy and transplanted sides was only possible in 9 patients and the result was the average of 5 trials. On the healthy side, the mean strength in individual patients was between 88.6 N and 383.2 N, while on the transplanted side only 12.4 N to 73.6 N, indicating a significant weakening of the bite force on the transplanted side of the mandible. In a similar way, the bite strength was also assessed by Bouchet et al. [15]. Using FlexiForce, they made 5 measurements for each patient and averaged the results, obtaining bite strength for the healthy side and the transplanted side. The strength for the healthy side was between 160 and 563 N, and for the transplanted side between 50 and 462 N, which also indicated a significant reduction in strength on the transplanted side of the mandible. Yamamoto [24] compared the strength of the occlusion before resection and reconstruction of the mandible with the condition after surgery. The results indicated a significant improvement in bite strength, however, the publication does not specify the measuring tool that was used to conduct the study. Chewing performance was also investigated in a relatively objective manner in the Zhang et al. [25] study. Patients underwent a Masticatory performance test consisting of chewing 5 g of peanuts, rinsing the mouth, spitting its contents into a measuring cylinder, and then sifting through a mesh with 2 mm holes. Based on the weight of the food residues on the mesh, the result of chewing efficiency was given as a percentage and indicated a significant deterioration in chewing performance after the procedure. In the same study, patients were also asked to subjectively assess chewing sensations using the University of Washington quality of life questionnaire [25]. It also confirmed a significant deterioration in chewing function. An important objective parameter assessed by the other researchers was also the range of mouth opening, which in the examined patients ranged from 2 to 9.5 cm [15, 17, 21, 23, 26–29]. Generally, these values indicated an improvement in the extent of oral opening compared to the state before surgery, but not in all patients [26]. The significance of the functioning of the mouth, mandible, and speech apparatus was also the subject of research by other authors, but their tools of assessment were mostly questionnaires and scales [30–38]. An interesting fact is a discrepancy among the assessments of patients after the procedure, ranging from excellent speech and swallowing function, through good and acceptable jaw function, to difficulties during chewing and swallowing food.

### Evaluation of the global function associated with the donor site

The authors of the selected study also performed a functional assessment of the patient related to the donor site. Most often it is an assessment of the function of the lower limb due to the fact of taking a graft from the fibula or iliac crest free flap. Among all selected articles, 4 presented a comprehensive description of the patients’ condition related to the possibilities of moving and performing activities of everyday life. It is worth noting that only in these publications did the authors use objective assessment tools such as a dynamometer, Footscan platform, or mechanographic platform. An extensive analysis of lower limb function can be found in the article by Rendenbach et al. [39], in which patients underwent mandibular reconstruction with fibula free flap. The subjects were subjected to the evaluation of jump mechanography, which included its height, speed, as well as maximum force, and peak power. The results of the analysis showed a significant reduction in the height of the jump. On the other hand, the examination of maximum peak strength and power showed a decrease in values in both donor and untreated limbs, but these differences were not significant. In the case of both limbs, there was also an increase in the number of motions in the anteroposterior direction in the one-leg standing, but most of these results were statistically insignificant. Significant imbalances were noted only in the anteroposterior direction. The range of motion of the ankle joint was clearly decreased in dorsal and plantar flexion. A year later, Rendenbach et al. [14] also analyzed the function of patients whose mandibular reconstruction was performed from the iliac crest free flap. Although the assessment of the subjects on the Esslinger Fitness Index scale, in jumping tests, the test of getting up from the chair, and the balance test showed no significant differences before and after the procedure. There was a significantly worse range of motion of the hip joint, limitation of mobility in the lumbar spine, as well as occasional limitations of everyday activities such as getting up from sitting or lying down, or playing sports. The subjects did not complain about limping and sensory disturbances. Xu et al. [40] assessed lower limb function before surgery and then 3, 6, and 9 months after surgery. Among other things, they investigated the peak torque/weight for the ankle joint using an isokinetic dynamometer at angular velocity of 30, 60, and 90 degrees per second. The results clearly indicated a significant decrease in torque of the plantar and dorsal flexion of the ankle joint. Electromyography examination was also performed on the patient's calf in the area of the superficial peroneal nerve. In some patients, the conduction velocity and response amplitude dropped by almost half. Also, the response peak latency was doubled. Gait analysis was also carried out using the Footscan platform reporting the occurrence of asymmetry and changes in pressure distribution during standing and movement. In statics, in the donor limb, a clear transfer of the pressure to the heel part of the sole was shown. On the other hand, in the second limb, a significant increase in metatarsal pressure was noted. The other authors focused mainly on collecting data from the subjective patients’ assessment, in which the vast majority did not report major complications associated with collection. Some patients had slight limitations in the performance of everyday activities or weakness of the donor limb [14, 39, 41].

### Other ailments evaluation

The authors of the selected articles made an additional assessment that also included other complaints reported by patients. They concerned both the craniofacial area and donor sites. These included sensory disturbances in the lips and graft area, or pain in the oral cavity [15–17, 20, 34, 42]. Some patients also experienced neuropathic pain or local pain in the limb, as well as sense weakness in the donor site area or complete loss of sense along the peroneal nerve [14, 20, 40, 41, 43–46]. In some cases, scar hypertrophy, cold sensitivity at the donor site, and femoral nerve palsy also occurred [13, 40, 47].

## Discussion

The basis for effective treatment of any patient with planned tumor resection and subsequent mandible reconstruction should be the ability of the evaluation and prediction not only the short- but also long-term effects of surgeries in the context of the patients’ functioning. The mandibular reconstruction procedure is an extremely complicated procedure itself which is constantly being improved, among others through the use of 3D planning technology or computer-aided design and manufacturing during reconstruction surgery (CAD-CAM) [15, 48, 49]. In addition to the development of treatment technology, the methods of patients’ function assessing should also be developed. Whatis worth mentioning is that the aim of the vast majority of studies published in the years 2015–2022 was to describe the course of reconstruction and assess the patient’s condition immediately after the procedure to detect early postoperative complications such as wound infection, correct healing, hematomas or fistulas, and not a long-term assessment of the function of the mouth, temporomandibular joint and donor site [50]. After analyzing the collected articles, in the assessment of the long-term functioning of patients, one can notice a tendency of the authors to take into account largely only subjective assessments of patients on the basis of scales and questionnaires. On one hand, this is a positive tendency, because it puts the patient's feelings first, but from a scientific point of view, it is unreliable and not very objective. There is a definite lack of studies that assess the patient's function using objective tools that give results in the form of specific numerical values that allow intragroup and intergroup comparisons. An example is the study of subjective feelings of patients (instead of making objective measurements) in the assessment of ankle instability or decrease in muscle strength [10]. However, the least discussed topic is the objective assessment of the temporomandibular joint. A possible reason is the lack of technologically advanced equipment that would enable an objective examination of the patient. For example, among the selected articles, only one team assessed bite strength using a dedicated device [22]. Other assessments were carried out using modified devices and adapted to a specific study or with the help of original tests [15, 46]. The assessment of the patient's function itself is certainly an extremely important task, but the fact of what will happen to this knowledge in the next stage of restoring the patient to full fitness seems equally important. Sakuraba et al. [22] and Bouchet et al. [15] indicated a significant weakening of the bite force on the reconstructed side of the mandible, while Rendenbach et al. [39] mentioned significantly reduced lower limb strength and gait problems in some transplant patients. For physiotherapists, this may be a field for work on improving the measured parameters, and ultimately it may also have its effect in improving patients' satisfaction with the chewing or movement function. Early postoperative assessment of the patient is particularly important due to the possibility of complications of the transplanted site associated with potential blood supply disorders or its necrosis [51, 52], but later in the postoperative period, physiotherapists could assess the holistic patient's function and, on this basis, begin to introduce adequate rehabilitation. Some studies encompass postoperative physiotherapy of the patient, including the improvement of the function of the oral cavity and temporomandibular joint, or the improvement of the function of the lower limb as a donor site [14, 27, 30, 41, 53]. Taking into account the above considerations and the goal set for physiotherapy, its planning should be based on an objective assessment both in terms of functional and morphological changes, not only within the transplanted fragment of the mandible and adjacent areas, but also within the donor site. This is due to the fact that many researchers and patients subjectively notice deficits in functioning after treatment. However, to address the existing limitations, an objective assessment should be carried out, which is lacking in the published literature. Taking into account the complaints reported by patients noted in the lists above it would be possible to propose as a standard: an objective assessment of structural changes within the foot (claw toe), assessment of changes in gait parameters, examination of TMJ function, examination of possible imbalances and distribution of the general center of mass, examination of strength and muscle mass. Evaluation of the above parameters, performed with validated measuring devices, could indicate direct improvement needs involving both TMJ function and the donor area.

## Summary

The review shows different strategies for the treatment of mandible tumors, the most popular of which were reconstructions using fibula free flap and iliac crest free flap and furthermore different methods of functional assessment resulting from measurement techniques within the face and the donor site. However, to a large extent, the authors devote attention to subjective assessments of the postoperative condition of patients, omitting in many cases an objective assessment. The analyzed literature also does not indicate clear directions of postoperative physiotherapy of patients, however, on its basis it can be assumed that patients should undergo comprehensive rehabilitation including improvement of the function of the jaw, TMJ, soft tissues of the head and neck, as well as activities related to the rehabilitation of the donor site. From the physiotherapy point of view, this creates real benefits for planning a comprehensive rehabilitation procedure involving the activation of the mandible, TMJ along with soft tissues, as well as the distant area, constituting donor sites for tissue acquisition. Some researchers assessed the function or facial aesthetics of their patients for a period ranging from six months to even 38 years after surgery [14, 16, 17, 19–27, 30, 32–35, 37, 40, 43–46, 53–55]. Such a long-term analysis may be important for physiotherapists in terms of reliable assessment of the patient's condition at individual stages of rehabilitation. Because of the fact that these treatments could be introduced, the quality of patients' lives will effectively improve, however, more research is needed in this area.

## Data Availability

Not applicable.
